# Hexokinase and phosphofructokinase activity and intracellular distribution correlate with aggressiveness and invasiveness of human breast carcinoma

**DOI:** 10.18632/oncotarget.4910

**Published:** 2015-08-07

**Authors:** Raquel G. Coelho, Isadora C. Calaça, Deborah M. Celestrini, Ana Helena P. Correia-Carneiro, Mauricio M. Costa, Patricia Zancan, Mauro Sola-Penna

**Affiliations:** ^1^ Laboratório de Enzimologia e Controle do Metabolismo (LabECoM), Departamento de Biotecnologia Farmacêutica, Faculdade de Farmácia, Universidade Federal do Rio de Janeiro, Rio de Janeiro, Brasil; ^2^ Instituto de Bioquímica Médica Leopoldo De Meis, Universidade Federal do Rio de Janeiro, Rio de Janeiro, Brasil; ^3^ Hospital Universitário Clementino Fraga Filho, Universidade Federal do Rio de Janeiro, Rio de Janeiro, Brasil; ^4^ Laboratório de Oncobiologia Molecular (LabOMol), Departamento de Biotecnologia Farmacêutica, Faculdade de Farmácia, Universidade Federal do Rio de Janeiro, Rio de Janeiro, Brasil; ^5^ Present address: Laboratório de Fisiologia Endócrina Doris Rosenthal (LFE), Instituto de Biofísica Carlos Chagas Filho, Universidade Federal do Rio de Janeiro, Rio de Janeiro, Brasil

**Keywords:** glycolysis, prognosis, breast cancer, treatment, diagnosis

## Abstract

Glycolytic enzymes, such as hexokinase and phosphofructokinase, have been reported to be upregulated in many cancer types. Here, we evaluated these two enzymes in 54 breast cancer samples collected from volunteers subjected to mastectomy, and the results were correlated with the prognosis markers commonly used. We found that both enzymes positively correlate with the major markers for invasiveness and aggressiveness. For invasiveness, the enzymes activities increase in parallel to the tumor size. Moreover, we found augmented activities for both enzymes when the samples were extirpated from patients presenting lymph node involvement or occurrence of metastasis. For aggressiveness, we stained the samples for the estrogen and progesterone receptors, HER-2, p53 and Ki-67. The enzyme activities positively correlated with all markers but Ki-67. Finally, we conclude that these enzymes are good markers for breast cancer prognosis.

## INTRODUCTION

Breast cancer is the most common malignant cancer type in women globally, costing billions of dollars per year for its treatment [1–4]. The success of the treatment is directly correlated to the stage of the cancer upon initial treatment, which depends on how early the cancer has been detected. However, one of the major problems associated with this cancer type is that the strong side effects of the treatment still cause severe discomfort to the patients. [5]. Therefore, breast cancer prevention and treatment programs aim to identify more efficient and precise diagnostic and prognostic markers, as well as targets for a treatment with milder side effects [5].

Approximately 80% of breast cancers are invasive, breaking though the ductal or glandular walls where they originated. Thus, these two types of invasive breast cancers are designated as Infiltrating Ductal Carcinoma (IDC; ∼80% of invasive breast cancers) and Invasive Lobular Carcinoma (ILC; ∼10% of invasive breast cancers), respectively [4]. According to the stage of invasion, breast cancers are classified as 0, I, II, III or IV, where 0 is the non-invasive stage and IV is when the cancer has invaded the lymph nodes and other tissues such as the lungs, liver, skin, brain, etc. [6]. In terms of prognosis, breast cancers are classified according to size (T), the involvement of lymph nodes (N) and the occurrence of distant metastasis (M). This TNM classification is widely used, attributing numbers according to the severity of the case [6]; T is classified from 0–4 where 0 is no evidence of a tumor, N is classified from 0–3 and M is classified as 0 or 1, following the same principle [6, 7]. Additionally, many other markers have been used to determine the prognosis of breast cancer, and among the commonly used markers are whether the tumors are negative or positive for the presence of the following proteins: progesterone and estrogen receptors (PR and ER, respectively), human epidermal growth factor receptor 2 (HER-2), the nuclear protein Ki-67 and the tumor suppressor p53 [8–10]. Normally, tumors containing receptors for the sexual hormones are fast growing, which worsens the prognosis [9]. The same principle is also valid for the presence of HER-2 [9, 11]. However, the presence of these receptors is also used to target drugs directly to tumors. For instance, tamoxifen and trastuzumab are directed toward ER and HER-2 positive cancers, respectively [12–14]. The most aggressive type of breast cancer is negative for PR, ER and HER-2 and is termed the triple negative [9, 11]. In addition, the presence of Ki-67 and/or the absence of p53 are strongly indicative of a highly proliferative and aggressive cancer [10, 15, 16].

To support the high rate of proliferation, cancers have adapted their metabolism so that they present a unique metabolic profile [16, 17]. Designated the ‘Warburg effect’, cancers consume large amounts of glucose through glycolysis, mostly producing lactate and even consuming an elevated amount of oxygen [18–24]. Oxygen is consumed due to the high activity of certain reactions in the citric acid cycle that are mainly converting glutamine into carbon skeletons (fatty acids, cholesterol, amino acids, etc.) for biomass production [24]. At the same time, glycolysis furnishes ATP to support metabolism as a whole [19, 22–24]. Many alterations allow these cells to present a high glycolytic profile. Among these alterations, we and other groups have devoted special attention to the intracellular distribution of the key glycolytic enzymes hexokinase (HK) and phosphofructokinase (PFK). Both enzymes present complex regulatory mechanisms, being activated and inhibited by many allosteric ligands. For instance, HK, the first enzyme in the glycolytic pathway, is inhibited by its product, glucose-6-phosphate (G6P). The inhibition of HK reflects on the decrease of glucose uptake by the cell [25]. Likewise, PFK, the second kinase in glycolysis, is inhibited by one of its substrates, ATP, when at concentrations higher than 1 mM [26, 27]. Since ATP is also one of the final products of glycolysis, an excess of this metabolite reflects on the inhibition of PFK and the whole glycolysis [28]. On the other hand, these enzymes can associate with other intracellular components, altering their catalytic rates and regulatory properties. For example, the activity of these enzymes is enhanced when HK binds to the voltage-dependent anion channel (VDAC) in mitochondria [25, 29] and when PFK binds to actin filaments (f-actin) in the cytoskeleton [28]. Moreover, these associations reflect on the loss of allosteric inhibition of HK by G6P and of PFK by ATP. It has been shown that the association of HK with VDAC and of PFK with f-actin, namely, particulate HK and PFK, are increased in tumors, compared to non-tumoral tissues [28, 30–40]. One hypothesis is that increased particulate HK and PFK is part of the mechanism that originates the ‘Warburg effect’ [41].

In the present study, we aimed to correlate the increased HK and PFK activity with the altered intracellular distribution of the prognosis markers for human breast cancers. We evaluated the enzymes in material (cancerous and non-cancerous tissues) collected from 54 donors subjected to mastectomy after cancer diagnosis. Our results reveal a strong correlation between the particulate fraction of the enzymes and the markers for the most aggressive cancer types.

## RESULTS

**Figure 1:**
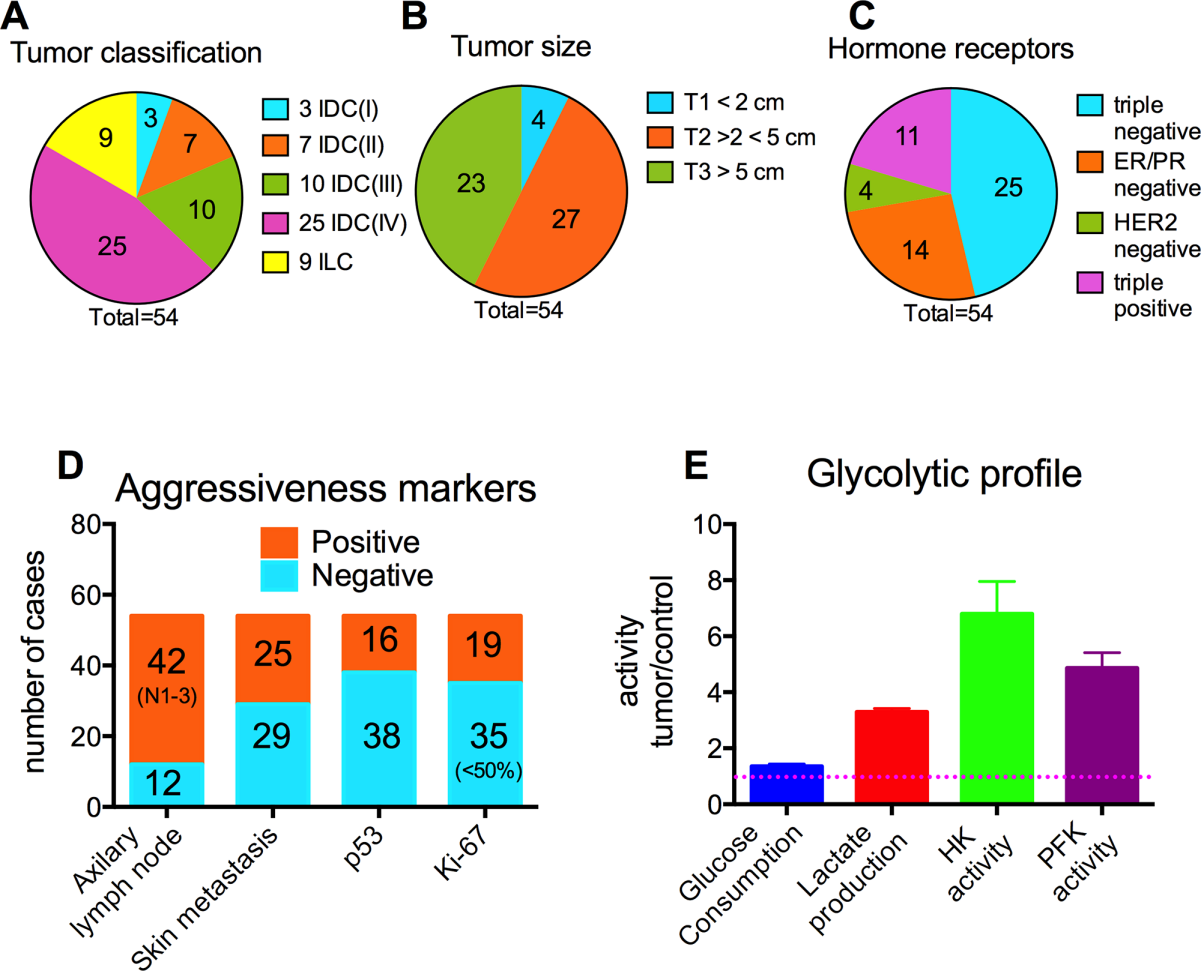
Profile of the breast carcinoma samples **Panel A.** Classification of the tumors. **Panel B.** Size classification according to TNM. **Panel C.** Responsiveness to the hormone receptors. **Panel D.** Aggressiveness markers. **Panel E.** Glycolytic profile. The glycolytic profile was investigated as described in the Materials and Methods. The plotted values are the mean ± standard errors of at least 40 samples. The dotted line is related to the control values, *i.e.,* when the ratio between the values for non-tumoral tissues and tumoral tissues is equal to 1. All the plotted values are significantly different from the values obtained with non-tumoral tissues (*P* < 0.05, two-way ANOVA, Sidak's post-test).

The current work has analyzed tumoral and non-tumoral (adjacent to tumoral and used as a control) breast tissues from 54 female volunteers (average age 62 ± 11 years) subjected to mastectomy at the major hospital of the Federal University of Rio de Janeiro (Hospital Universitário Clementino Fraga Filho – HUCFF/UFRJ) between 2007 and 2009. All the volunteers signed the free and informed consent terms prior to surgery and were aware of all procedures concerning the samples. All samples were morphologically and histologically classified according to the TNM classification, as well as for the presence of ER, PR Her-2, p53 and Ki-67. Additionally, glycolytic markers, including glucose consumption rate, lactate production rate, HK activity and PFK activity, were measured in tumor cells and compared to the non-tumoral counterparts from the same patient. The results are summarized in Figure 1. Among the tissues collected, 83% were diagnosed as IDC and 17% as ILC. IDC samples were further sub-classified as stage I (7%), II (16%), III (22%) and IV (55%) according to the invasion level of the cancer (Figure 1A). Regarding tumor size, due to the samples characteristics, we subdivided the samples into three groups, where 7% were less than 2 cm (T1), 50% were more than 2 cm but less than 5 cm (T2) and 43% were more than 5 cm (T3; Figure 1A). Additionally, 46% of the tumors were triple negative (Figure 1C), and 78% and 46% of the patients presented compromised axillary lymph nodes and metastasis on skin, respectively (Figure 1D). Furthermore, 70% and 35% of the tumors were negative for p53 and positive (> 50% staining) for Ki-67, respectively (Figure 1D). The overall classification analysis revealed that most of the tumors analyzed presented a very aggressive profile. Moreover, compared to the non-tumoral tissues excerpted from the patients, tumors presented elevated glycolytic markers, (Figure 1E) such as glucose consumption rate (35% higher; *P* < 0.05), lactate production rate (3.3-fold higher; *P* < 0.05), HK activity (3.5-fold higher; *P* < 0.05) and PFK activity (2.0-fold higher; *P* < 0.05).

**Figure 2:**
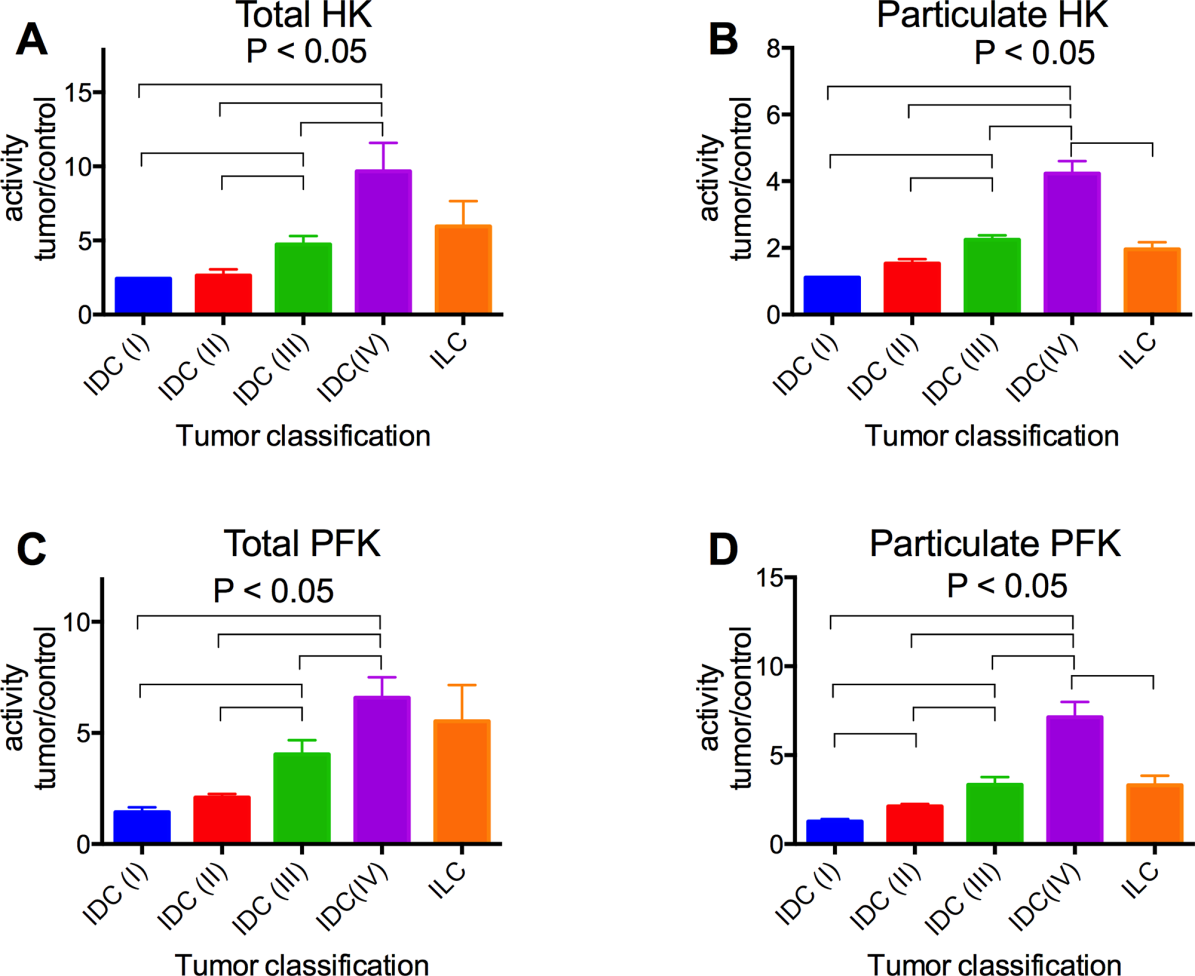
HK and PFK activities of the tumor samples separated with regard to tumor classification Tumor classification was performed according to invasiveness as described in the Materials and Methods. HK and PFK activity in the total and particulate fractions was analyzed as described in the Materials and Methods. **Panel A.** Total HK activity. **Panel B.** Particulate HK activity. **Panel C.** Total PFK activity. **Panel D.** Particulate PFK activity. The plotted values are the means ± standard errors, and the numbers of cases are in accordance with the results presented in Figure 1. Statistical analyses were conducted using ANOVA and Sidak's post-test.

**Figure 3:**
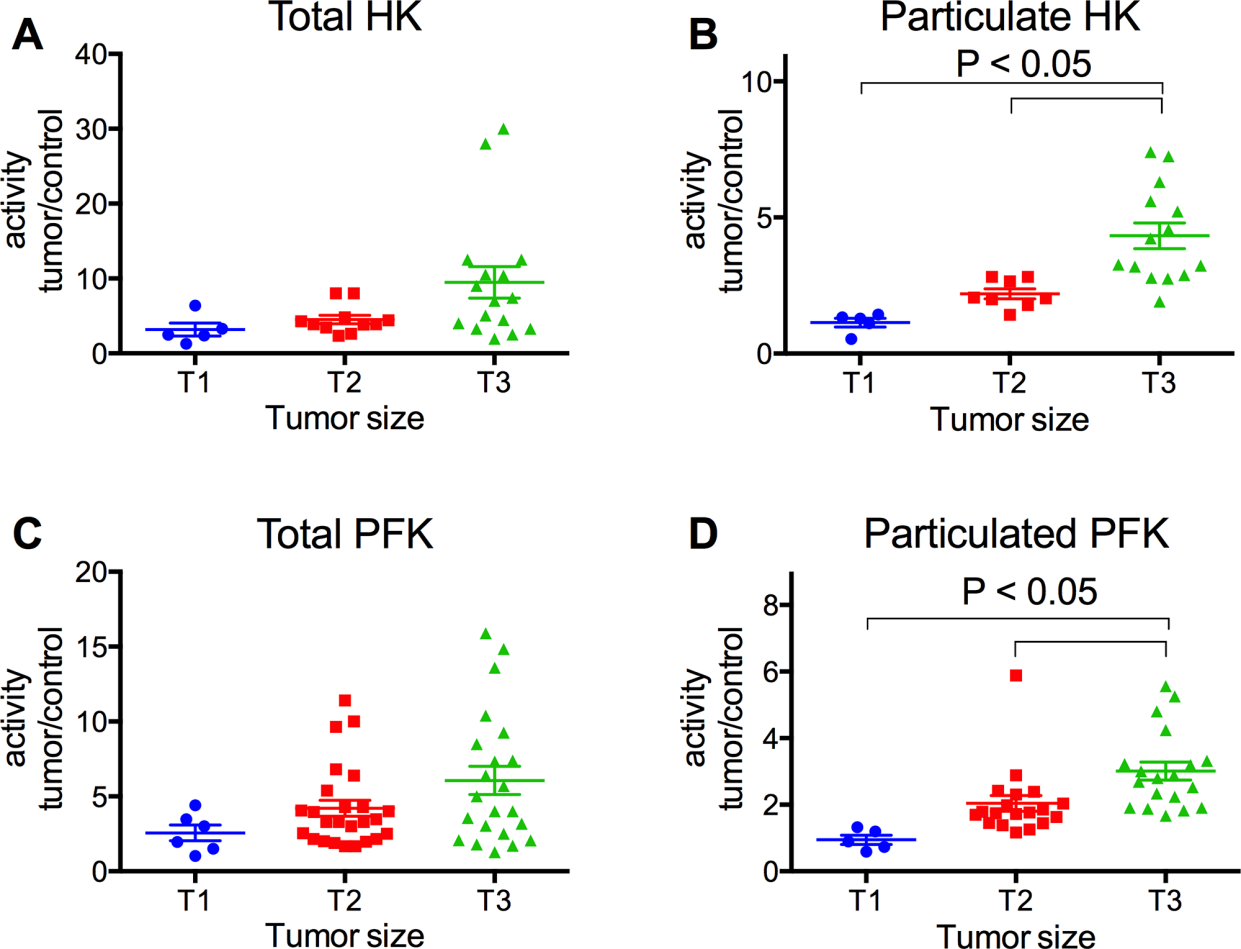
HK and PFK activities of the tumor samples separated with regard to tumor size Tumor size groups, T1, T2 and T3 were separated as described in the Materials and Methods. HK and PFK activity in the total and particulate fractions was analyzed as described in the Materials and Methods. **Panel A.** Total HK activity. **Panel B.** Particulate HK activity. **Panel C.** Total PFK activity. **Panel D.** Particulate PFK activity. The plotted values are the mean of enzyme activity evaluated in triplicate for each tumor sample. Statistical analyses were conducted using ANOVA and Sidak's post-test.

**Figure 4:**
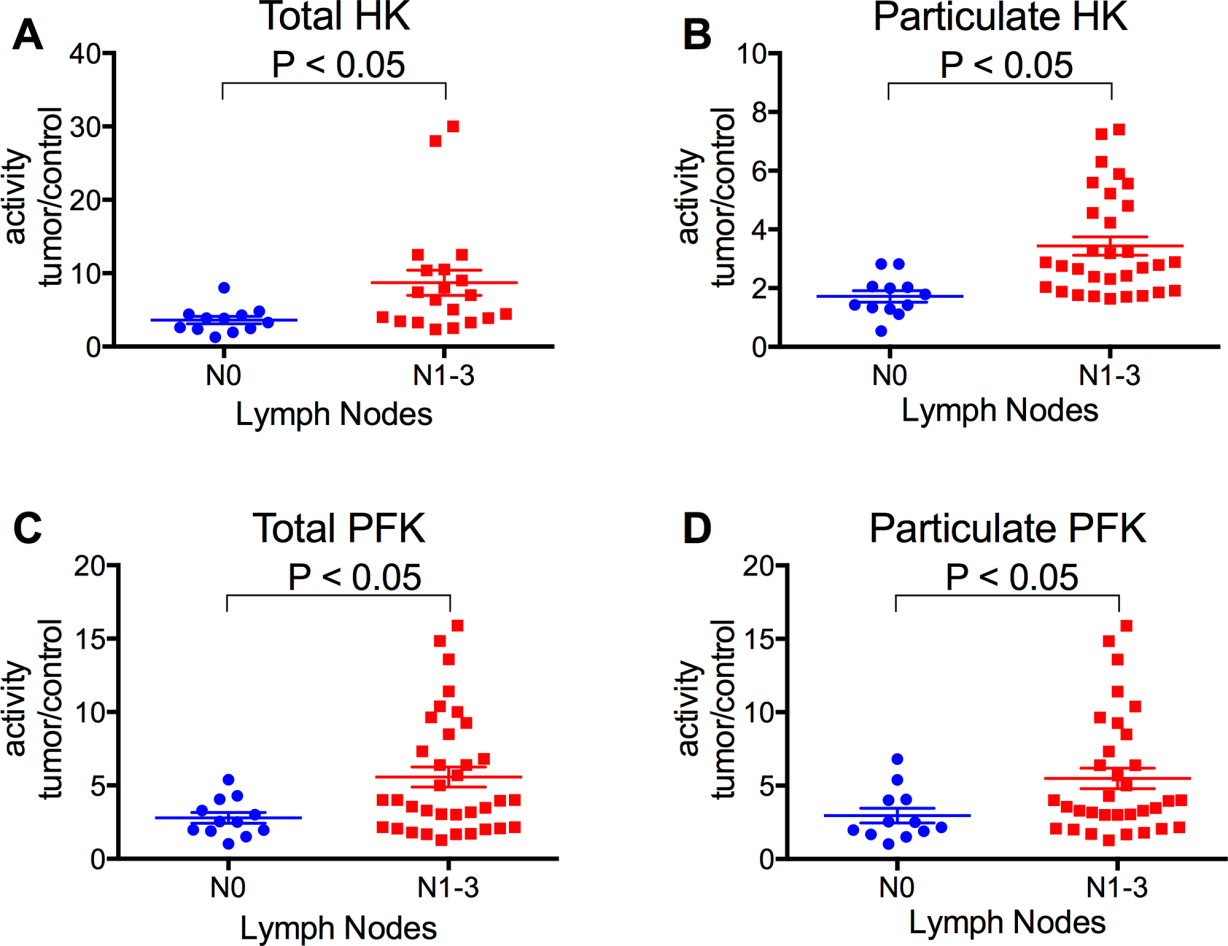
HK and PFK activities of the tumor samples separated with regard to the presence of cancer cells in the lymph nodes Lymph nodes were analyzed as described in the Materials and Methods. HK and PFK activity in the total and particulate fractions was analyzed as described in the Materials and Methods. **Panel A.** Total HK activity. **Panel B.** Particulate HK activity. **Panel C.** Total PFK activity. **Panel D.** Particulate PFK activity. The plotted values are the mean of enzyme activity evaluated in triplicate for each tumor sample. Statistical analyses were conducted using ANOVA and Sidak's post-test.

**Figure 5:**
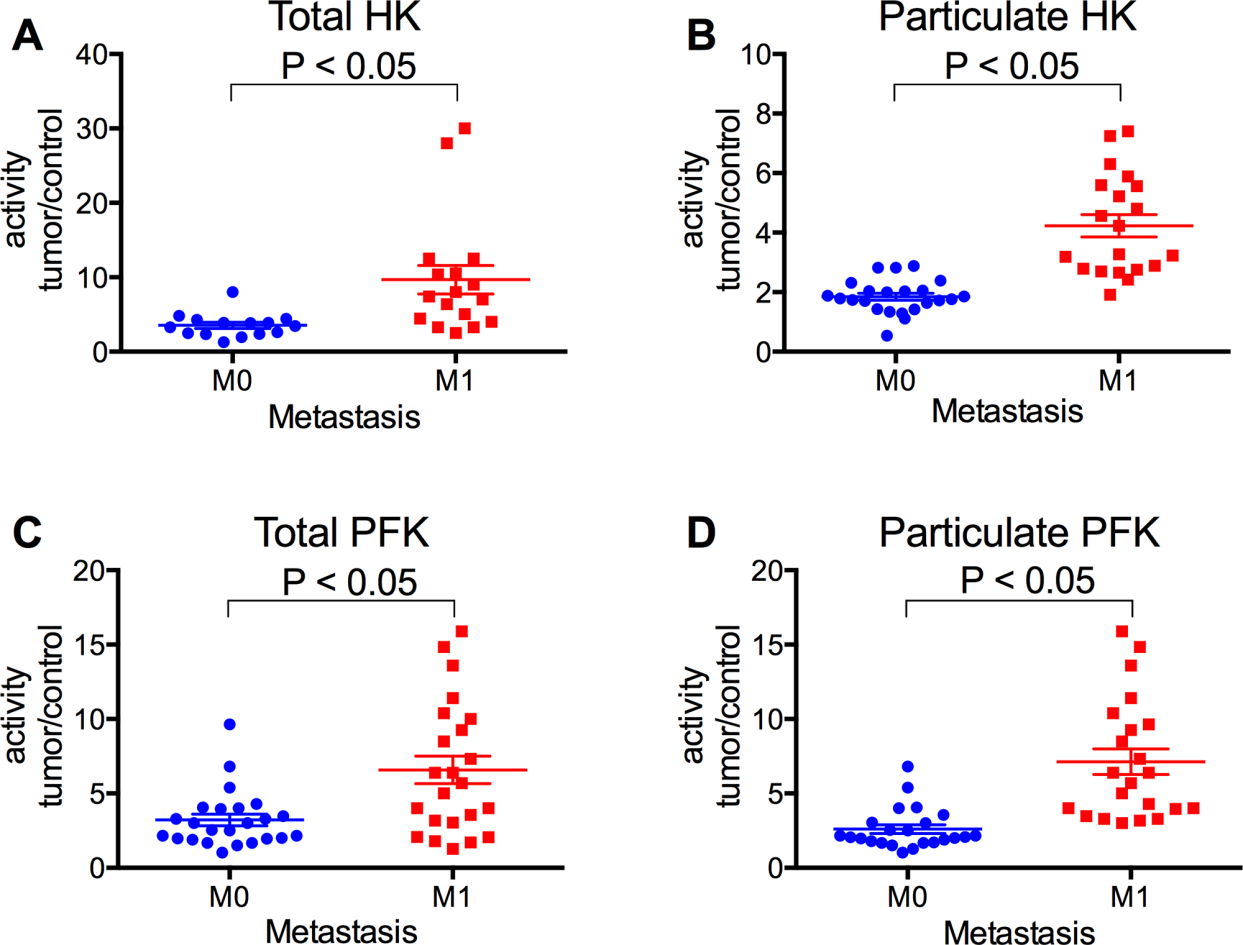
HK and PFK activities of the tumor samples separated with regard to the occurrence of metastasis Samples were classified as M0 or M1 by the presence of skin metastasis in the patients as described in the Materials and Methods. HK and PFK activity in the total and particulate fractions was analyzed as described in the Materials and Methods. **Panel A.** Total HK activity. **Panel B.** Particulate HK activity. **Panel C.** Total PFK activity. **Panel D.** Particulate PFK activity. The plotted values are the mean of enzyme activity evaluated in triplicate for each tumor sample. Statistical analyses were conducted using ANOVA and Sidak's post-test.

The activity of HK and PFK was further analyzed in terms of the ratio of the activities of the enzymes in tumor samples relative to non-tumor samples, according to the stage of invasion and TNM classification of the tumors. We analyzed not only the total activity but also the particulate activity of both enzymes. The IDC tumors in stage IV presented much higher HK and PFK activities (total and particulate activities), compared to the others (Figure 2A–2D). Indeed, the enzyme activities among the IDC groups increased proportionally to the invasiveness of the tumors (I < II < III < IV). ILC samples were also analyzed, and they tended to present lower enzyme activities than IDC samples (IV), confirming the proportionality of the invasiveness. There was also a positive correlation between the tumor sizes and the activity of the particulate fraction of both HK and PFK (Figure 3B and 3D, respectively). However, no statistically significant differences were observed in the total activity of the enzymes (Figure 3A and 3C). The activities of both enzymes, either total or particulate, were higher when there is the involvement of lymph nodes (Figure 4) or the occurrence of skin metastasis (Figure 5).

**Figure 6:**
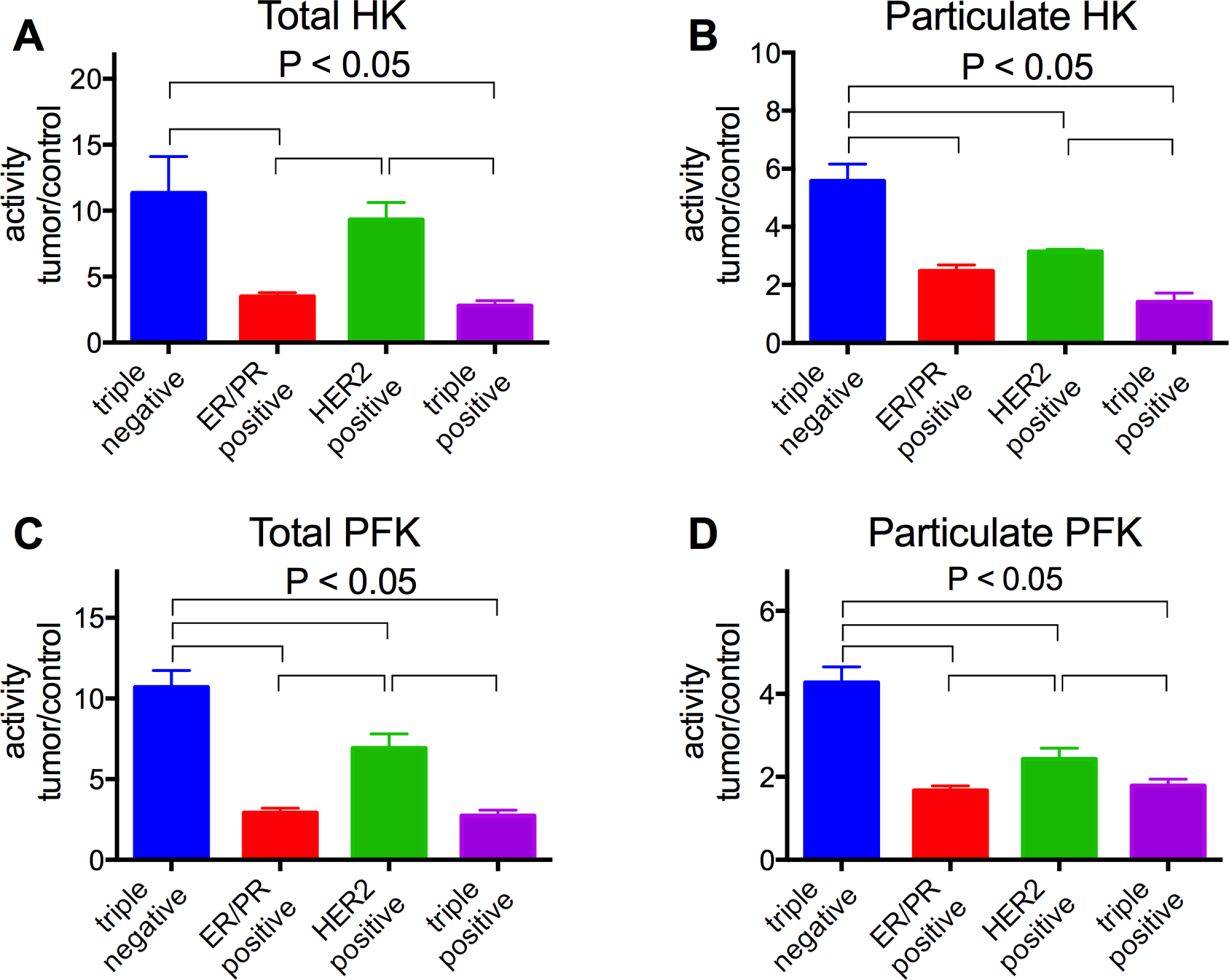
HK and PFK activities of the tumor samples classified according to the expression of hormone receptors Tumor samples were stained for ER, PR and HER-2 as described in Materials and Methods. HK and PFK activities in the total and particulate fractions were analyzed as described in the Materials and Methods. **Panel A.** Total HK activity. **Panel B.** Particulate HK activity. **Panel C.** Total PFK activity. **Panel D.** Particulate PFK activity. Plotted values are the means ± standard errors, and the numbers of cases are in accordance with the results presented in Figure 1. Statistical analyses were conducted using ANOVA and Sidak's post-test.

**Figure 7:**
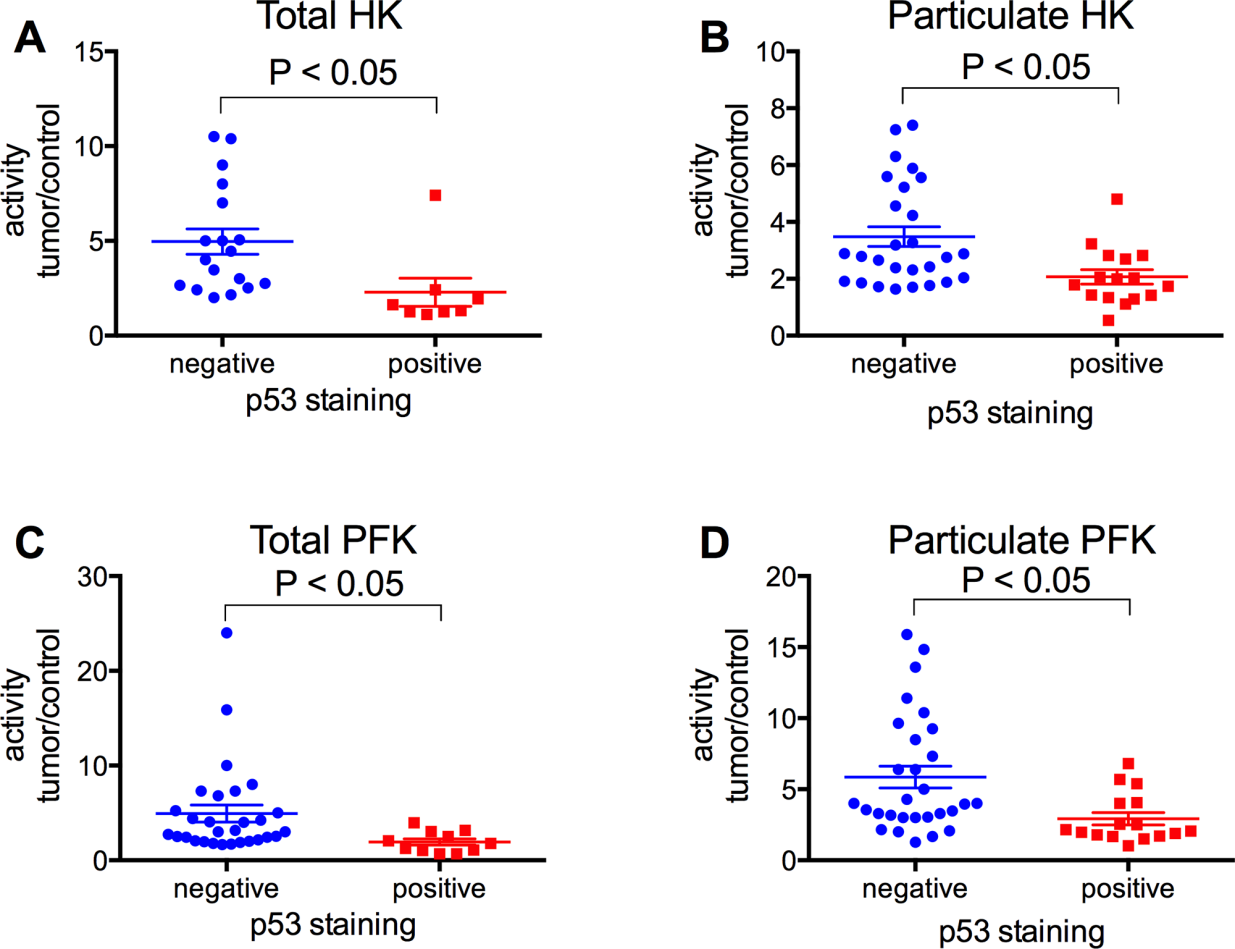
HK and PFK activities of the tumor samples classified according to the expression of p53 Tumor samples were stained for p53 as described in Materials and Methods. HK and PFK activity in the total and particulate fractions was analyzed as described in the Materials and Methods. **Panel A.** Total HK activity. **Panel B.** Particulate HK activity. **Panel C.** Total PFK activity. **Panel D.** Particulate PFK activity. The plotted values are the mean of enzyme activity evaluated in triplicate for each tumor sample. Statistical analyses were conducted using ANOVA and Sidak's post-test.

**Figure 8:**
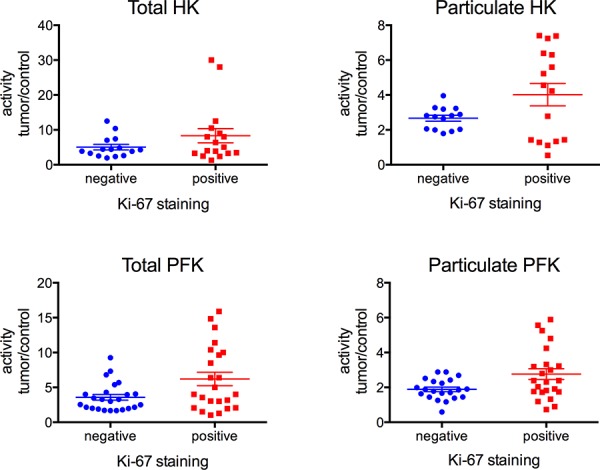
HK and PFK activities of the tumor samples classified according to the expression of Ki-67 Tumor samples were stained for Ki-67 as described in Materials and Methods. HK and PFK activity in the total and particulate fractions was analyzed as described in the Materials and Methods. **Panel A.** Total HK activity. **Panel B.** Particulate HK activity. **Panel C.** Total PFK activity. **Panel D.** Particulate PFK activity. The plotted values are the mean of enzyme activity evaluated in triplicate for each tumor sample. Statistical analyses were conducted using ANOVA and Sidak's post-test.

According to the presence of hormone receptors in the tumors, our results clearly show that the triple negative samples present higher activities of HK and PFK (total and particulate), compared to all the other samples (Figure 6A–6D, blue bars). The triple negative was lower than the HER-2 positive samples (Figure 6A) only for total HK activity. Indeed, the HER-2 positive tumors presented HK and PFK activities higher than the triple positive and the PR/ER positive samples, and only the particulate HK activity presented no difference between HER-2 positive and PR/ER positive samples (Figure 6B). The triple positive and the PR/ER positive samples presented lower activities of the enzymes and were not different between themselves for all of the analyses (Figure 6A–6D). Comparing the samples that were negative and positive for p53 staining, it was clear that the p53 negative tumors presented higher activities of both enzymes in total and particulate fractions (Figure 7). However, we found no differences in the enzymes comparing Ki-67 negative and positive tumors (Figure 8).

## DISCUSSION

The increased glycolytic profile associated with a high production of lactate, even in the presence of an appropriate supply of oxygen, is one of the hallmarks of cancer [16, 18–20, 22]. This unique altered metabolic profile is the result of several changes and/or mutations in proteins, mainly enzymes, transcription factors/controllers and receptors, that control the metabolic flux [41]. Among these alterations, we have reported that PFK, one of the major regulatory glycolytic enzymes, has altered activity [31, 35], expression patterns [32] and intracellular distributions in cancers [30–32, 34, 35]. Moreover, we found that the intracellular distribution alteration, *i.e.,* the association with f-actin within the cytoskeleton, is more pronounced in metastatic cancers compared to non-metastatic cancers [34, 35]. Although the association between PFK and f-actin is one of the physiological mechanisms of enzyme activation by many stimuli [28, 42–54], the detachment of PFK from f-actin has proven to be lethal for cancer cells but non-lethal to non-tumor cells [31, 34, 36–38, 54]. Following the same pattern, HK associates with mitochondria as an activating mechanism, and this association is more pronounced in cancers [36, 37, 40, 55]. The detachment of HK from mitochondria decreases the viability of tumor cells but has no effect on non-tumor cells [55–57]. Therefore, it has been previously proposed that the increased activity of HK and PFK, as well as their increased particulate fractions, *i.e.,* f-actin-bound PFK or mitochondria-bound HK, is characteristic of tumor cells [16, 18–21, 24, 32, 41, 55, 57, 58]. Therefore, the current work investigated the correlation between the total activity of these two glycolytic enzymes and their particulate fraction activity, as well as the most commonly used prognostic markers for human breast cancer.

Most of the breast cancer samples we obtained (91%) were diagnosed as infiltrating ductal carcinoma (IDC), which is compatible with the frequency of this type of cancer [4, 59]. These samples were further divided by the stage of invasion following the most acceptable guidelines [7, 59] among four groups: I, II, III and IV, where I is the least invasive and IV presents metastasis. Our data suggests that HK and PFK activities correlate with the invasiveness of the cancer (Figure 2). The IDC (IV) presented a much higher activity than the other samples, which was also confirmed when the samples were separated as M0 and M1 (non-metastatic or metastatic, respectively; Figure 5). Because tumor invasiveness correlates with tumor size [2, 10], it is not surprising that we also found a correlation between enzyme activity and tumor size (Figure 3). In spite of the lack of statistically significant differences among the total enzyme activity when separated by tumor size, the tendency is clearly observed (*P* = 0.068 for total HK, Figure 3A and 0.057 for total PFK, Figure 3C; two-way ANOVA), and the increase is supported by the particulate enzymes activity results (Figure 3B and 3D). Moreover, an additional marker for breast tumor aggressiveness is the presence of tumor cells within the axillary lymph nodes [2, 10]. When our samples were classified according to this marker, we observed an increased activity of both HK and PFK, in the total and particulate fractions (Figure 4). Taken together, the results presented in Figures 2, 3, 4 and 5 suggest that HK and PFK activity directly correlates to breast cancer tumor aggressiveness.

Different biochemical markers such as ER, PR, HER-2, p53 and Ki-67 have been used to determine the prognosis of breast cancers [2]. However, the conclusion about the relevance and significance of the results of these markers is still unclear [60]. However, it is clear that the triple negative breast cancer lacking ER, PR and HER-2 is the cancer presenting the worst prognosis, and usually presents a more aggressive behavior and poorer outcome when compared to the others [61]. The triple negative samples from the current work also presented the higher enzyme activities, either in total and particulate fractions, compared to the ER/PR positive, HER-2 positive or the triple positive samples (Figure 6). Only for the total HK activity is the triple negative not significantly different from the HER-2 positive (Figure 6A, *P* = 0.079, two-way ANOVA, Sidak's post-test). Although not significant, the results suggest a tendency because the activity for the triple negative and HER-2 positive samples is different for the HK particulate fraction (Figure 6B). Moreover, the statistical analysis was potentially compromised because we only analyzed 4 HER-2 positive samples. However, the fact that the HER-2 positive samples, which are considered to present a worse prognosis than the ER/PR positive and triple positive tumors, presented the second highest HK and PFK activity strengthens the correlation between the enzyme activity and the prognosis for breast cancer patients. The same conclusion can be reached when the samples are separated into p53 negative and positive, which present worse and better prognoses, respectively [62]. With regard to HK and PFK activity, p53 negative samples presented the higher activity (Figure 7), resulting in, again, the worse outcome. However, we found no correlation between HK and PFK activities and Ki-67 expression in our samples (Figure 8). Although recommended as a prognostic marker, the relevance and significance of this marker is still under scrutiny, and this marker has been used only in association with other markers [63, 64].

In conclusion, the present work presents irrefutable evidence for the correlation between the activity of HK and PFK and the prognosis of breast cancer patients. Further extensive work with patients must be conducted to evaluate how the activity of these enzymes may predict the outcome. However, it is undeniable that their activity is directly correlated to the aggressiveness and invasiveness of breast cancer.

## MATERIALS AND METHODS

### Materials

ATP, glucose and fructose-6-phosphate (F6P) were purchased from Sigma Chemical Co. (St. Louis, MO, USA). ^32^Pi was purchased from Instituto de Pesquisas Energéticas e Nucleares (São Paulo, Brazil). [γ-^32^P]ATP was prepared according to [65]. All protein content measurements were performed as described by [66].

### Volunteer tissues and data collection

All tissues were obtained from female donors along with the written consent of the patients undergoing mastectomy at Hospital Universitário Clementino Fraga Filho (HUCFF/UFRJ), Rio de Janeiro, Brazil. Tumor and control tissues from the same donors were removed during surgery. After histological analysis for the classification and grading of tumors, samples were immediately frozen in liquid N_2_ and stored until further use. This project was developed after the approval of the National Ethical Committee (CONEP—approval protocol 1897.0.000.197-06).

### Tissue fractionation

Tissue fractionation was performed as previously described [31, 35]. After N_2_ withdrawal, tissues were homogenized with a Polytron homogenizer in a buffer containing 50 mM Tris–HCl (pH 7.4), 0.25 M sucrose, 20 mM KF, 0.2 mM 2-mercaptoethanol and 0.5 mM phenylmethanesulfonyl fluoride (PMSF). Homogenized tissues were centrifuged for 5 min at 100 g (4°C) for separation of cellular debris and non-digested tissues. The resultant supernatant, called total homogenate (TH), represents the total enzyme activity. The TH was centrifuged for 15 min at 27000 g (4°C), and the supernatant was centrifuged again for 45 min at 120.000 g (4°C). The resulting high-speed supernatant was used to evaluate the soluble fraction enzyme activity, while the respective pellet was resuspended in the original volume with the same buffer and used to analyze the particulate fraction enzyme activity.

### Immunohistochemistry

The most representative tumor tissue block was chosen from each sample, and 5 μm sections were attached to poly-L-lysine-coated slides for immunohistochemical staining. The tissue sections were deparaffinized in xylene, rehydrated in alcohol, and immersed in distilled water. The sections were then boiled for ten minutes in citrate buffer solution (10 mM, pH 6.0) in a microwave oven 3 times for epitope retrieval in staining, and the standard streptavidin-biotin immunoperoxidase method was used for immunostaining with Ki-67 antigen, HER-2, ER, PR and p53 according to [67]. The sources and dilutions of these antibodies and the epitope retrieval methods are listed in Table 1. Bound antibodies were detected using Envision TM mouse or rabbit (Dako, USA) secondary antibodies after a 45 min incubation at room temperature. Samples were considered HER-2 positive if they scored 3+ according to the appropriate guidelines [63].

**Table 1:** Details of immunohistochemical analysis

Primary antibody to	Clone/ Source	Dilution	Epitope retrieval method
Estrogen receptor (ER)	R;SP1/Thermo Scientific	0.38889	Pressure cooker, 9 min
Progesterone receptor (PR)	M;PgR636/Thermo Scientific	0.73611	Pressure cooker, 9 min
c-erbB2/HER-2/neu	R;SP3/Thermo Scientific	0.11111	Microwave oven
p53 protein	M; DO-7/Dako	1.91667	Pressure cooker, 8 min
Ki-67 antigen	M; MIB1/Dako	0.45833	Pressure cooker, 8 min

Pressure cooker: citrate buffer (pH 6) (Tender Cooker, Nordic Wave, USA). Microwave: citrate buffer (pH 6), 15 min (Eletrolux, 900 W).

### Enzymatic activity assays

HK and PFK activities were measured by the method described in [68] with modifications introduced by [69, 70]. The reaction media contain 50 mM Tris–HCl (pH7.4), 5 mM MgCl_2_, 1 mM [γ-^32^P]ATP (4 μCi/nmol), 50 μg/ml of the tissues extracts (considering their protein content), and 1 mM fructose-6-phosphate or 5 mM glucose, for PFK and HK activities, respectively. The reaction was stopped after increasing reaction times by the addition of a suspension of activated charcoal in 0.1 M HCl and 0.5 M mannitol. After centrifugation, the supernatant products were analyzed using a liquid scintillation counter. The signals from the appropriate blanks (in the absence of fructose-6-phosphate or glucose) were measured and subtracted from all measurements to account for ATP hydrolysis. The catalytic rate was calculated by linear regression analysis of the amount of products formed vs. reaction time.

### Statistics

Statistical analyses were performed using Prism 6 (GraphPad Software Inc., La Jolla, CA, USA). A two-way ANOVA using Sidak's post-test was applied for all analyses, and the results were considered significantly different when *P* < 0.05.
